# A Molecularly Imprinted Polymer on a Plasmonic Plastic Optical Fiber to Detect Perfluorinated Compounds in Water

**DOI:** 10.3390/s18061836

**Published:** 2018-06-05

**Authors:** Nunzio Cennamo, Girolamo D’Agostino, Gianni Porto, Adriano Biasiolo, Chiara Perri, Francesco Arcadio, Luigi Zeni

**Affiliations:** 1 Department of Engineering, University of Campania “Luigi Vanvitelli”, via Roma 29, 81031 Aversa, Italy; fra.arc91@gmail.com (F.A.); luigi.zeni@unicampania.it (L.Z.); 2 Copernico S.r.l., Via Monte Hermada 75, 33100 Udine, Italy; girolamodag@gmail.com (G.D.); porto@copernicon.it (G.P.); biasiolo@copernicon.it (A.B.); chiaraperri91@gmail.com (C.P.); 3 IREA-CNR, Via Diocleziano 328, 80124 Napoli, Italy

**Keywords:** surface plasmon resonance (SPR), plastic optical fiber (POF), molecularly imprinted polymer (MIP), perfluorooctanoate (PFOA), perfluorooctanesulfonate (PFOS), perfluorinated alkylated substances (PFAs), optical sensors

## Abstract

A novel Molecularly Imprinted Polymer (MIP) able to bind perfluorinated compounds, combined with a surface plasmon resonance (SPR) optical fiber platform, is presented. The new MIP receptor has been deposited on a D-shaped plastic optical fiber (POF) covered with a photoresist buffer layer and a thin gold film. The experimental results have shown that the developed SPR-POF-MIP sensor makes it possible to selectively detect the above compounds. In this work, we present the results obtained with perfluorooctanoate (PFOA) compound, and they hold true when obtained with a perfluorinated alkylated substances (PFAs) mixture sample. The sensor’s response is the same for PFOA, perfluorooctanesulfonate (PFOS) or PFA contaminants in the C_4_–C_11_ range. We have also tested a sensor based on a non-imprinted polymer (NIP) on the same SPR in a D-shaped POF platform. The limit of detection (LOD) of the developed chemical sensor was 0.13 ppb. It is similar to the one obtained by the configuration based on a specific antibody for PFOA/PFOS exploiting the same SPR-POF platform, already reported in literature. The advantage of an MIP receptor is that it presents a better stability out of the native environment, very good reproducibility, low cost and, furthermore, it can be directly deposited on the gold layer, without modifying the metal surface by functionalizing procedures.

## 1. Introduction

PFAs have been widely used for the last four decades in many industrial sectors and their dispersion in water has been recognized as highly dangerous for eco-systems, biodiversity and human health. The EU directive 2013/39/UE lists PFAs among the priority substances to be completely eliminated within the next 20 years, thus making this issue extremely urgent.

PFOA and PFOS are the most extensively investigated PFAs, because human exposure can occur through different pathways, although dietary intake seems to be their main route of exposure [1].

These contaminants are very persistent and refractory to different biological and chemical treatments and their presence in the environment can give rise to toxicity and bio-accumulative effects, particularly to mammalian species.

Immunotoxic effects of perfluorinated alkylated substances to cellular systems and animals are widely demonstrated [2,3], and different epidemiologic research studies have shown the potential effects of these chemical compounds on various human immune diseases.

The conventional proposed analytical methods are based on chromatographic techniques coupled with mass spectrometry [4,5,6,7,8]. Furthermore, sensors based on electrochemical and colorimetric approaches have also been described [9]. All of the mentioned methods are time-consuming, expensive and they often require a complicated pre-treatment step. In order to beat these drawbacks, it is needed to find a rapid, simple and sensitive method for the detection of perfluorinated alkylated substances.

In PFOA, PFOS or total PFAs detection, a very attractive perspective is the use of a platform based on optical fibers for fast in situ and/or remote-controlled detection. For different applications, biosensors in optical fibers allow for remote sensing and for reduced dimensions and price of the whole sensor system [10,11,12,13]. In particular, several review papers describe plasmonic optical fiber sensor platforms and their applications [14,15,16,17,18,19].

On this line of argument, we exploited a low cost surface plasmon resonance (SPR) sensor platform, based on plastic optical fibers (POFs) [20], together with a novel biomimetic polymer for the detection of PFOA/PFOS in an aqueous medium. POFs are particularly advantageous due to their easily handling and installation procedures, large diameter of the fiber (a millimetre or more), low-cost and simplicity in manufacturing [21,22,23]. In a previous work, Cennamo et al. [24] built an SPR-POF sensor based on bio-receptors obtaining an LOD of 224 ppt. In this work, a new synthetic receptor, specifically designed to recognise C4 to C12 PFAs, is used with the same SPR-POF platform reaching a better LOD (130 ppt). This result could be considered of interest when compared to the detection limit of PFAs obtained by using different approaches, as reported in Oughena et al. [25] and Trojanowicz et al. [26] or Cennamo et al. [27].

The molecular imprinting technique is a convenient tool for the preparation of molecular-recognition materials characterized by good chemical stability and selectivity. Molecular imprinted polymers are biomimetic materials imprinted with a template molecule for the purpose of retaining a memory of that specific analyte (or a specific class of molecules). MIPs exhibit many favourable aspects with respect to bio-receptors, such as an easier and faster preparation, the possibility of application outside the laboratory, for example under environmental conditions, a longer durability. Moreover, the advantage of MIPs is that they can be directly deposited on a flat gold surface by a spin coater machine without modifying the surface (functionalization and passivation), as needed for bio-receptors [24].

## 2. Materials and Methods

### 2.1. Materials

Reagents: (Vinylbenzyl)trimethylammonium chloride [CAS 26616-35-3] (VBT), 2,2-azobisiso-butyronitrile [CAS 78-67-1] (AIBN), 1*H*,1*H*,2*H*,2*H*-perfluorodecyl acrylate [CAS 27905-45-9] (PFDA) were obtained from Sigma–Aldrich (Saint Louis, MO, USA) and used without any further purification. Ethylene glycol dimethacrylate [CAS 97-90-5] (EDMA) (Sigma–Aldrich) were distilled under vacuum prior to use in order to remove stabilizers.

A certified reference material is also used to prepare the standards for dose/response curve: CRM ref n. CPA 98FE.1.N.1.5 (CPAchem Ltd., Stara Zagora, Bulgaria) a mixture of 11 components (perfluoropentanoic acid [CAS 2706-90-3], undecafluorohexanoic acid[CAS 307-24-4], perfluoroheptanoic acid [CAS 375-85-9], perfluorooctanoic acid [CAS 335-67-1], perfluoro-nonanoicacid [CAS 375-95-1], perfluorodecanoic acid [CAS 335-76-2], perfluoroundecanoic acid [CAS 2058-94-8], nonafluoro-1-butanesulfonic acid [CAS 375-73-5], perfluorooctanoate sulfonic acid [CAS 1763-23-1], heptafluorobutyric acid [CAS 375-22-4], tricosafluorodecanoic acid [CAS 375-22-4]).

All other chemicals were of analytical reagent grade. The solvent was deionised water. Stock solutions were prepared by weighing the solids and dissolving in ultrapure water (Milli-Q^®^, Merck KGaA, Darmstadt, Germany).

### 2.2. Production of MIP for PFOA and NIP

The prepolymeric mixture for MIP was prepared according to a previously optimized procedure, based on ammonium perfluorooctanoate (FPO-NH4) as the template, VBT and PFDA as the functional monomers, EDMA as the cross-linker and AIBN as the radicalic initiator. The reagents were mixed at the following molar ratio 1(Template):4(VBT):5(PFDA):50(EDMA). The mixture was uniformly dispersed by sonication (visually homogeneous milky solution). Deionised water was added to dissolve all reagents (volume ratio H_2_O:EDMA = 1:17.5). Finally, the AIBN was added to the solution in non-stoichiometric ratio. Also, a second monomeric solution was prepared. The composition was the same as previously described but without adding any template, in order to obtain an NIP (non-imprinted polymer).

### 2.3. Optical Sensor Platform

**Figure 1:**
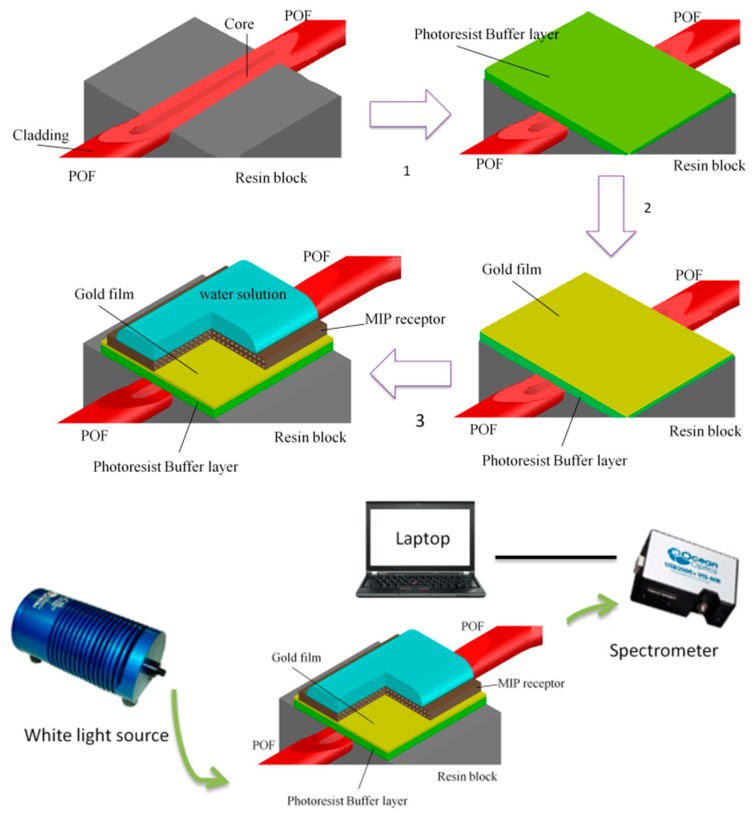
Production steps for realizing an SPR sensor in a D-shaped POF with an MIP receptor and outline of the experimental setup.

The surface plasmon resonance (SPR) sensor is based on a D–shaped POF with an optical buffer layer (Microposit S1813, MicroChem Corp., Westborough, MA, USA) between the exposed POF core and the thin gold film. This optical platform is realized by removing the cladding of POF (along half circumference), spin coating the buffer layer on the exposed core and, finally, sputtering the gold film (see Figure 1). The plasmonic sensing area is about 10 mm in length. In the visible range of interest, the buffer layer (the photoresist Microposit S1813) presents a higher refractive index than the one of the POF core. This optical buffer layer improves the performances of the SPR sensor [20]. The size of the POF is 980 μm of core (PMMA) and 10 μm of cladding (fluorinated polymer), whereas the multilayer on D-shaped POF presents a thickness of the buffer layer of about 1.5 μm and a thin gold film of 60 nm.

As shown in Figure 1, the planar gold surface can be employed for depositing the MIP receptor layer, as we will explain in the following section. In this case, the selective detection of the analyte is possible. The outline of all the production steps, from the polishing step to the MIP deposition, with the experimental setup are summarized in Figure 1.

### 2.4. The Experimental Equipment

The simple and low-cost experimental setup is based on a halogen lamp (HL–2000–LL, Ocean Optics, Dunedin, FL, USA), as the light source, the SPR-POF sensor and a spectrometer (FLAME-S-VIS-NIR-ES, Ocean Optics, Dunedin, FL, USA) connected to a PC. The wavelength emission range of the halogen lamp goes from 360 nm to 1700 nm, whereas the spectrometer presents a detection range from 350 nm to 1023 nm (see Figure 1).

The SPR curves, along with data values, were displayed online on the computer screen and saved with the help of the advanced software provided by Ocean Optics. The SPR transmission spectra, normalized to the reference spectrum, achieved with air as the surrounding medium, are obtained using the Matlab software (MathWorks, Natick, MA, USA). The Hill fittings of the experimental values are obtained through OriginPro software (Origin Lab. Corp., Northampton, MA, USA).

The resin block of the SPR-POF sensor is fixed on the optical table. Every time, after that the SPR curve in air (reference spectrum) is acquired, the measurements are obtained without moving the chip. If the chip sensor is moved, the reference spectrum must be acquired again.

### 2.5. Deposition of the MIP and NIP Layer

The MIP and the NIP layers were deposited as hereafter described. The planar sensing area (the gold surface) was washed with ethanol, then dried in a thermostatic oven at 60 °C prior to deposition of the polymer layers (MIP or NIP).

For both layers, MIP and NIP, 50 μL of the prepolymeric mixture were dropped over the sensing region (SPR surface) of the chip and spun for 80 s at 1500 rpm.

For both the polymer layers, the thermal polymerization was then carried out for 16 h at 74 °C.

The obtained polymeric film was washed and the template molecule was extracted, leaving the imprinting sites free for rebinding.

The washing and extraction procedures were characterized by two steps.

In the first step, the MIP and NIP layers were washed with 96% v/v ethanol in order to remove not-polymerized monomers residue.

In a second step, the template was extracted from MIP by washing with HCl solution (2% *w*/*w*) and 96% *v*/*v* ethanol.

The first step is conducted flushing 5 mL of ethanol on the platform and second step flushing 1.5 mL of HCl solution, 5 mL of ethanol, 1.5 mL of HCl and 5 mL of ethanol. Finally, the sensor was flushed with deionised water and dried at room temperature.

### 2.6. Binding Experiments

The experimental results were collected by the SPR-POF-MIP sensor and the previously illustrated measurement setup. After each addition of the sample (solution with different concentration of the analyte), we have used a standard measuring protocol based on the following three steps: first, incubation step for chemical-interaction between analytes and MIP receptor (for 10 min at room temperature); second, washing step with water (blank); third, recording step for the spectrum, when water (blank) is present as the bulk. This protocol is necessary in order to measure the shift of the resonance determined by the specific binding (analyte/receptor interaction) on the sensing surface, and not by the changes of the bulk refractive index or by non-specific binding between gold surface and analyte.

Finally, we have obtained different results exploiting a platform based on SPR-POF-NIP sensor and the same measurement set-up as above. In particular, we deposited the NIP layer on the same D-shaped POF platform. In this case, we used the same values of the PFOA concentrations and the same three steps used in the binding experimental (SPR-POF-MIP sensor): incubation step (10 min at room temperature); washing step (with water); recording step for the spectrum, when water is present as the bulk.

## 3. Results

### 3.1. PFAs Detection

**Figure 2:**
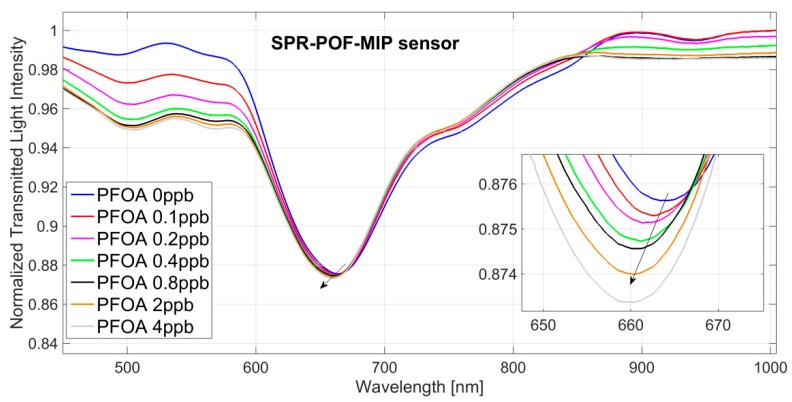
SPR spectra obtained at different concentrations of PFOA in water solution (0–4 ppb) by an SPR-POF-MIP sensor. Inset: zoom of the resonance wavelengths.

Figure 2 shows the transmission spectra of the SPR-POF-MIP normalized to the reference spectrum (spectrum achieved with air as the surrounding medium), obtained by incubating solutions at increasing concentrations of PFOA in water solution (range 0–4 ppb).

In an SPR-POF platform when the refractive index at the gold–dielectric interface increases, according to SPR phenomenon theory, the resonance wavelength is shifted to the right [20].

When an MIP receptor layer is present on the gold film, the penetration of the analyte in the MIP layer produces a change (usually an increase) in the resonance wavelength due to the variation (usually an increase) of the refractive index at the interface between the MIP layer and the gold film.

As shown in Figure 2, in this case, the resonance wavelength is shifted to smaller values by increasing the concentration of PFOA in water solution. A shift like this means that, when the PFOA interacts with the MIP receptor, the refractive index value of the MIP layer decreases. This phenomenon is also present when the PFOA interacts with the antibody (bio-receptor) on the same SPR-POF platform [24].

This effect is related to the chemical composition of the perfluorinated compounds. We verified this behaviour measuring the refractive index at high concentrations of PFOA in water solutions, by an Abbe refractometer. We found that when the PFOA concentration greatly increases in the water, the refractive index of the water solution slightly decreases.

Therefore, in order to exclusively measure the shift of the resonance determined by the specific binding (analyte/MIP) on the sensing surface, and not by the changes of bulk refractive index, we used all the three previously described steps: incubation step, washing step with water, and spectrum recording step when the water (blank) is present as the bulk.

**Figure 3:**
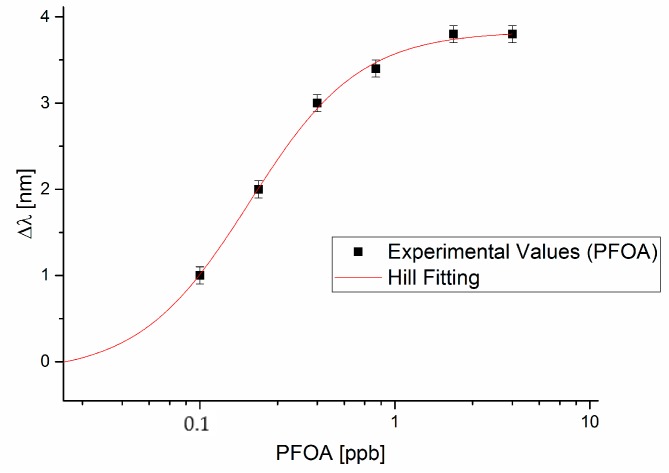
Plasmon resonance wavelength variation (∆λ), with respect to the blank, versus the concentration of PFOA (ppb) and Hill fitting to the experimental values, in semi-log scale.

Exploiting the resonance wavelengths plotted in Figure 2 and Figure 3 reports the resonance wavelength shift, with respect to the blank (PFOA 0 ppb), versus PFOA concentration, in a semi-log scale, along with the Hill fitting to the experimental data. Each experimental value is the average of 5 subsequent measurements and the respective standard deviations (error bars), are shown as well.

**Figure 4:**
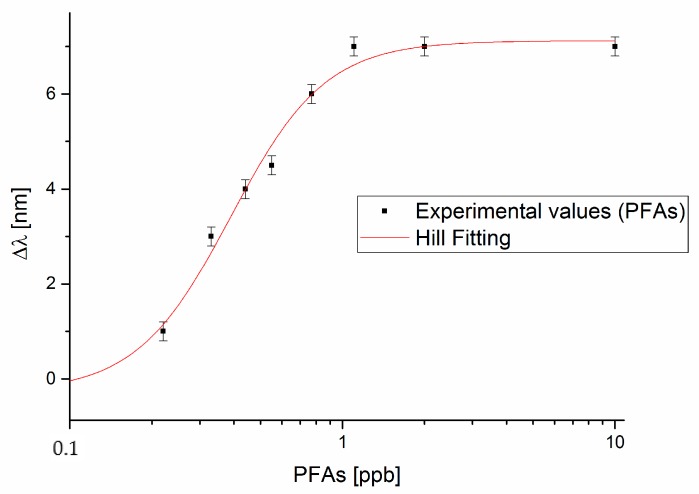
Plasmon resonance wavelength variation (∆λ), with respect to the blank, versus the concentration of PFAs (ppb) and Hill fitting to the experimental values, in semi-log scale.

Figure 4 shows the dose-response curve, with the Hill fitting to the experimental data, acquired when the PFAs compounds are present in a mix standard (a certified reference material containing 11 different PFAs (C4–C11)). As it will be shown in Discussion section, the performances obtained in the PFOA or PFAs detection are the same. The total resonance wavelength variation (∆λ_max_) in Figure 4 is a bit different with respect to that reported in Figure 3, because when the dimension/weight of the analyte changes the refractive index variation in the MIP layer changes.

### 3.2. No Binding Detection

**Figure 5:**
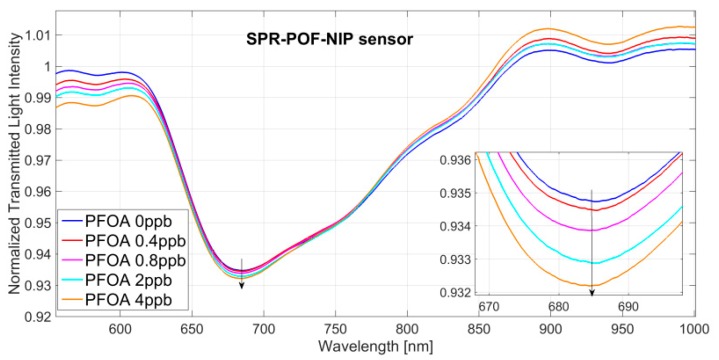
SPR spectra obtained at different concentrations of PFOA in water solution (0–4 ppb) by an SPR-POF platform with an NIP layer. Inset: zoom of the resonance wavelengths.

In order to verify the non-specific binding between the sensing layer and analyte, the response of SPR-POF-NIP sensor was tested. Figure 5 shows the SPR curves at different concentrations of PFOA (0–4 ppb). When the PFOA concentration increases, the shift of the resonance wavelength is not present.

## 4. Discussion

### 4.1. Analysis of the Dose-Response Curve

The Hill fittings reported in Figure 3 and Figure 4 are obtained through OriginPro software and the parameters, obtained with the associated standard errors, are listed in Table 1.

The Hill’s equation, used in the fitting of data, is reported in the following:

(1) $$\Delta \lambda_{c}\;=\lambda_{c}-\lambda_{0}=\;\Delta \lambda_{max}\;\cdot \;\frac{c^{n}}{\left(K^{n}\;+\;c^{n}\right)}$$

where *c* is the analyte concentration, *λ_c_* is the resonance wavelength at the concentration *c*, *λ*_0_ is the resonance wavelength at zero concentration (blank), ∆λ_max_ is the maximum value of ∆*λ_c_* (calculated by the saturation value minus the blank value), whereas *n* and *K* are the Hill constants and they can also have a physical meaning, as it will be discussed below. Standardization curves like the one reported in Figure 3 and Figure 4 are commonly used for chemo and biosensors, and their physical meaning can be related to the fact that the absorption takes place by combination at specific sites, when the number of receptor sites available for the combination with the substrate is limited [28]. In that case, the adsorption takes place according to the Langmuir absorption isotherm, as previously reported in case of a different MIP based sensor [21]. Moreover, the parameter n in the Langmuir model is equal to 1, which has been here experimentally found.

In this section, we present a comparison between the experimental results obtained in this work and the results obtained with the same SPR D-shaped POF platform but with a bio-receptor (antibody) for PFOA [24]. From Equation (1), it is possible to notice that, if n ≈ 1 and at low concentration, i.e., at *c* much lower than *K*, the dose-response curve is linear, with sensitivity ∆λ_max_/K, defined as the “sensitivity at low concentration”, as shown in Equation (2):

(2) $$\lambda_{c}-\lambda_{0}=\Delta \lambda_{c}=\frac{\Delta \lambda_{max}}{K}\cdot c$$

From Table 1, for the SPR-POF-MIP sensor we obtained the sensitivity at low concentration and the LOD, for both PFOA and PFAs, in water solution. Table 2 reports the obtained “sensitivity at low concentration” and the LOD (for PFOA and PFAs detection). The parameters, obtained by the same SPR-POF platform with an antibody for PFOA [24], are also reported in Table 2 for comparison purposes. The LOD can be calculated as the ratio of three times the standard deviation of the blank and the sensitivity at low concentration (Δλ_max_/K) [23].

Table 2 clearly shows that the same performance obtained with an SPR-POF platform with a bio-receptor for PFAs is obtained by this SPR-POF-MIP sensor. As previously stated, the advantage of MIPs is that they can be directly deposited on the gold surface, without modifying the surface. Moreover, the MIPs are synthetical receptors presenting a number of favorable features for sensing in comparison to bio-receptors, such as a better stability out of the native environment, reproducibility and low cost.

### 4.2. Surface Characterization by SPR Approach

**Figure 6:**
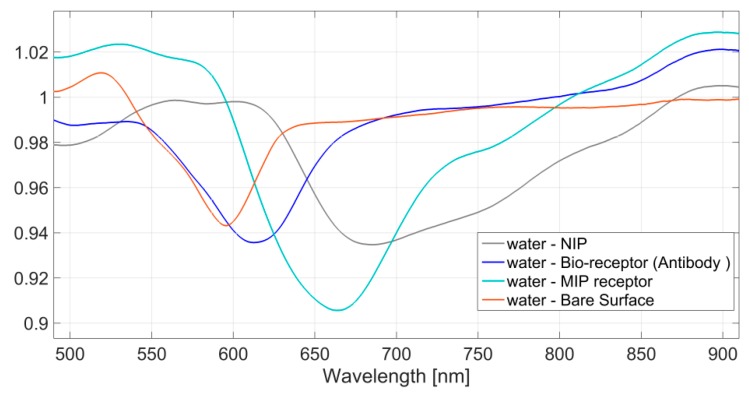
SPR spectra acquired in the presence of water on different surfaces: bare, with a bio-receptor, with an MIP receptor and with an NIP layer.

In optical sensors based on SPR in a D-shaper POF, as previously described, when the refractive index at the gold–dielectric interface increases, the resonance wavelength is shifted to the right [21,22,23]. This can be exploited to monitor the deposition process of the receptor layer (MIP receptors or Bio-receptors), since the presence of the receptor on the gold film produces an evident change in the resonance wavelength due to the variation of the refractive index at the interface between dielectric layer and the thin gold film. Figure 6 shows the resonance wavelength, when the water is present as the bulk, in the following cases: the gold surface without a receptor layer (bare surface), the gold surface with a bio-receptor for PFOA [24], the gold surface with an MIP receptor and, finally, the gold surface with an NIP receptor. The experiments were performed at room temperature and each sample was incubated 10 minutes before acquiring the signal. A shift is clearly shown in Figure 6, the refractive index of the NIP is larger than the MIP’s one, while the MIP refractive index itself is larger than the bio-receptor’s one.

Therefore, the immobilization of the bio-receptor or the deposition of the MIP/NIP layer on the sensor surface (gold film) can be directly monitored by SPR measurements exploiting the same optical platform.

In the future, we will characterize the MIP/NIP layer by SEM; meanwhile, we have shown how preliminary information about the MIP/NIP layer on the gold surface can be estimated by SPR curves (by their shape and position of the dip).

## 5. Conclusions

We have designed, realized and tested a novel MIP receptor for PFAs sensing by a plasmonic fiber sensor. This chemical optical sensor system is selective and able to sense very low concentrations of PFAs, with an LOD down to 0.13–0.15 ppb. The performances of the proposed system are comparable to those obtained by a specific antibody for PFOA deposited on the same optical platform, but it exhibits the classic advantages of MIP receptors: low-cost and very good reproducibility, better stability out of the native environment and the possibility of directly depositing it on the gold surface, without modifying the surface itself.

## Figures and Tables

**Table 1:** Hill parameters (SPR-POF-MIP sensor).

	λ_0_ [nm]		∆λ_max_ [nm]		K		n		Statistics	
Analyte	Value	Standard Error	Value	Standard Error	Value	Standard Error	Value	Standard Error	Reduced Chi-Sqr	Adj. R-Square
PFOA (Figure 3)	−0.138	0.941	3.833	0.108	0.179	0.060	1.537	0.411	1.075	0.995
PFAs (Figure 4)	−0.277	0.922	7.120	0.264	0.389	0.069	2.506	0.707	11.238	0.984

**Table 2:** PFOA and PFAs detection in water by an SPR-POF-MIP sensor and, for comparison, PFOA detection by [24] (an SPR-POF sensor with a bio-receptor).

Receptor	Parameters	Value
MIP Receptor	Sensitivity at low c of PFOA [nm/ppb]	22.14
	Sensitivity at low c of PFAs [nm/ppb]	18,99
	LOD [ppb] (3 × standard deviation of blank/ sensitivity at low c of PFOA)	0.13
	LOD [ppb] (3 × standard deviation of blank/ sensitivity at low c of PFAs)	0.15
Antibody [24]	Sensitivity at low c of PFOA [nm/ppb]	29.82
	LOD [ppb] (3 × standard deviation of blank/sensitivity at low c of PFOA)	0.24
